# A condom uterine balloon device among referral facilities in Dar Es Salaam: an assessment of perceptions, barriers and facilitators one year after implementation

**DOI:** 10.1186/s12884-020-2721-9

**Published:** 2020-01-13

**Authors:** Oluwakemi Adegoke, Sandra Danso-Bamfo, Margaret Sheehy, Vincent Tarimo, Thomas F. Burke, Lorraine F. Garg

**Affiliations:** 1 Department of Emergency Medicine, Division of Global Health Innovation, Massachusetts General Hospital, 125 Nashua St, Suite 910, Boston, MA 02114 USA; 2 Harvard T.H. Chan School of Public Health, Boston, MA USA; 3 Muhimbili National Hospital, Dar es Salaam, Tanzania; 4 Harvard Medical School, Boston, MA USA

**Keywords:** Postpartum hemorrhage, Uterine balloon tamponade, Implementation, Maternal mortality, Developing countries

## Abstract

**Background:**

Postpartum hemorrhage (PPH) is the leading cause of maternal death in Tanzania. The Every Second Matters for Mothers and Babies- Uterine Balloon Tamponade (ESM-UBT) device was developed to address this problem in women with atonic uterus. The objective of this study was to understand the barriers and facilitators to optimal use of the device, in Dar es Salaam Tanzania 1 year after implementation.

**Methods:**

Semi-structured interviews of skilled-birth attendants were conducted between May and July 2017. Interviews were recorded, coded and analyzed for emergent themes.

**Results:**

Among the participants, overall there was a positive perception of the ESM-UBT device. More than half of participants reported the device was readily available and more than 1/3 described ease and success with initial use. Barriers included fear and lack of refresher training. Finally, participants expressed a need for training and device availability at peripheral hospitals.

**Conclusion:**

The implementation and progression to optimal use of the ESM-UBT device in Tanzania is quite complex. Ease of use and the prospect of saving a life/preserving fertility strongly promoted use while fear and lack of high-level buy-in hindered utilization of the device. A thorough understanding and investigation of these facilitators and barriers are required to increase uptake of the ESM-UBT device.

## Background

Nearly 300,000 women die annually during pregnancy with postpartum hemorrhage (PPH) the leading cause worldwide [1]. Ninety-nine percent of these deaths occur in low-income countries [2]. In Tanzania, the maternal mortality ratio is 398 per 100,000 live births with hemorrhage and hypertensive disorders accounting for most of these deaths [3]. Although there are several risk factors for postpartum hemorrhage such as high parity, multiple gestations, polyhydramnios, prolonged and augmented labor, and pre-eclampsia [4] the majority of cases of PPH are unpredictable [5].

Uterotonics are recommended in the initial management of PPH. However, if hemorrhage is refractory to uterotonic agents, uterine balloon tamponade and surgical interventions such as uterine artery ligation, compression sutures, and/or hysterectomy may be employed [6]. Uterine balloon tamponade is non-invasive and appears able to avert the need for surgical intervention in the majority of women with uncontrolled PPH from atonic uterus [7–10]. In a systematic review, Tindell et al. reported that in the thirteen studies UBT effectively controlled postpartum hemorrhage in 234 of 241 cases, with average resolution of hemorrhage in 4 to 15 min. Complication rates were low in the use of all types of UBT, with no reported cases of uterine rupture and no increased risk of infection [10].

Since commercially available uterine balloon tamponade devices are expensive and often not available in low resource settings [11], the Division of Global Health and Innovation at the Massachusetts General Hospital developed an ultra-low-cost uterine balloon tamponade device known as Every Second Matters for Mothers and Babies- Uterine Balloon Tamponade (ESM-UBT). The ESM-UBT package consists of the ESM-UBT device, a PPH clinical management pathway, and a training module (Table 1). Over the past 10 years, the ESM-UBT device has been implemented in collaboration with in-country partners across more than 14 countries [12, 13], including four referral hospitals in Dar es Salaam, Tanzania. Prior research demonstrated that in this setting, women with uncontrolled PPH from atonic uterus who had ESM-UBT devices placed, survived 97% of the time overall and nearly 100% of the time when placed before advanced shock [14].

**Table 1:** ESM-UBT training curriculum

• Contents of device: syringe with a Luer lock valve, condom, Foley catheter with cotton string	
• Training and curriculum materials: PPH-UBT job aid checklist, a wall poster, PPH clinical pathway guide, a trainer’s flipchart, and a learner’s booklet	
• Trainees were instructed to use the UBT within the context of the established national protocol for PPH, which first included active management of the third stage of labor: • uterine massage • emptying the bladder • breastfeeding (if feasible) • identifying and treating perineal or cervical tears • administering prophylactic oxytocin and/or misoprostol (or other uterotonics if available) • manual removal of the placenta and blood clots • placement of the uterine balloon should occur if these interventions fail	
• Training-of-trainers model: the training program was a participatory, skills-based training that used hands-on PPH scenarios and simple maternal uterine models made of local components (e.g. a pillow surrounding a plastic water bottle, which represented a uterus within an abdomen)	
• Trainees were representative maternal health providers from each facility and were brought to a regional location for initial training.	
• These providers then returned to their facilities and trained staff with the target of ensuring that at least 85% of maternity providers in each facility achieved competence in UBT placement (defined as ‘online’ status).	
• All facilities received one wall chart checklist per delivery room, two manuals for PPH management and UBT use, and an adequate number of pre-deviced ESM-UBT kits (i.e. device and checklist).	

While the ESM-UBT device is both low cost and efficacious [13–15], changing health care provider behavior can be complex and challenging [16–20]. There is, however, a growing body of literature that seeks to identify implementation approaches that drive optimal provider performance and quality overall care [16, 18–22]. Tailoring interventions to address known determinants of practice has been shown to influence health care provider practice [23–26] and a number of authors have emphasized the importance of identifying barriers in order to strengthen implementation efforts [16, 17, 26, 27]. Given the challenges with changing health care provider practice patterns and the potential benefit of tailored interventions, it is important to identify those factors that lead to and impede uptake of the ESM-UBT device in order to adequately address them during implementation efforts. This study, which involved interviewing OBGYNs and skilled birth attendants 1 year after ESM-UBT was implemented at their hospitals in Dar es Salaam, Tanzania, was conducted to better understand facilitators and barriers to ESM-UBT device uptake and provider perceptions.

## Methods

All staff that attended births, including OBGYN specialists, nurses, midwives, physician assistants and non-specialist physicians, were invited to attend initial ESM-UBT trainings beginning in March 2016, across four hospitals in Dar Es Salaam, Tanzania. During that calendar year, these four hospitals performed a total of 46,072 deliveries. Trainings involved reviewing causes of postpartum hemorrhage, uterotonics, and treatment of postpartum hemorrhage, focusing primarily on the setup and utilization of the ESM-UBT device (Table 1). Facilities were officially ‘online’ when 85% of their staff had been trained. At least two refresher in-service trainings were also held at each facility. Following the trainings, use of the ESM-UBT device was considered standard practice for uncontrolled PPH from atonic uterus at the participating referral hospitals. A total of 29 OBGYN specialists, 141 medical officers and 297 midwives/nurses were trained on the ESM-UBT device through 22 training sessions.

In May and June of 2017, two external physician researchers (MS and OA) conducted semi-structured interviews of obstetrician-gynecologists, nurse-midwives, medical officers and post-graduate doctors employed at two of the four online hospitals (one tertiary and one district hospital). Providers were eligible for interview if they had participated in at least one ESM-UBT training module and had managed at least one case of uncontrolled PPH since that training. Convenience sampling of staff who satisfied inclusion criteria was employed due to the participants’ work schedules. All current staff who satisfied the inclusion criteria were then contacted by phone to determine their availability for an in-person interview. Verbal informed consent was obtained from participants prior to beginning the interviews. The researchers obtained phone numbers of individuals currently working at the two hospitals and contacted each person to determine if they were available for an interview. Interviews were audio recorded and transcribed verbatim. Investigators interviewed providers from diverse training backgrounds until thematic saturation was reached. Three researchers (OA, MA, SDB) independently coded the interview transcripts, reconciled any differences in coding, and identified themes that emerged from the interviews. A data codebook (Additional file 1) was created by author consensus through an iterative process. Data were coded using NVivo 11 (QSR International, Doncaster, VIC, Australia).

## Results

Twenty-five health workers were interviewed, nine of which were from Amana District Hospital and sixteen from Muhimbili National Hospital. Twelve of the interviewees were nurse midwives, six OBGYNS, six postgraduate doctors, and one a general practitioner. Nine were male and sixteen females. Ages ranged from 26 to 50 years. Sixteen (64%) of the interviewed health care workers reported greater than 5 years of experience. Several themes emerged and are described below:

### Perceptions and attitudes

All participants reported positive attitudes toward the ESM-UBT device while 13 additionally reported that their colleagues had positive things to say about the ESM-UBT device. A minority of doctors (three) stated their colleagues expressed a need for more education or “convincing” regarding the utility of the ESM-UBT device. All providers from both facilities reported that patient perceptions of the ESM-UBT device were overwhelmingly positive. Seven providers described fear as a barrier to UBT use. They feared being blamed for incorrect identification of the cause of hemorrhage and that placement of an ESM-UBT device could contribute to a delay in definitive intervention such as hysterectomy. They also expressed concern that they could be penalized for performing an intervention (UBT) about which their supervising consultants were unfamiliar. Two obstetricians described that hemorrhaging women were commonly transferred from other facilities in critical condition. They believed that this urgent situation may have led providers to perform procedures that they were more accustomed to and comfortable with such as supra-cervical hysterectomies. In addition, two OBGYN postgraduates stated that some supervising senior OBGYNs were resistant to ‘new’ conservative management modalities and appeared less interested than other providers in the ESM-UBT trainings.

### ESM-UBT device

Nearly one-third of those interviewed described that the ESM-UBT device was easy to use. One doctor mentioned that it was a simpler and quicker procedure than a hysterectomy while other interviewed providers reported they did not find it difficult to use the ESM-UBT device after the initial training. One provider stated that the time required to set up the ESM-UBT device was too long and suggested that a pre-assembled device might be easier to use.

### Training and knowledge

More than half the respondents stated they first heard about the ESM-UBT device during the initial training session and that the training program facilitated use in hemorrhaging women. Four midwives felt more comfortable using an ESM-UBT device after completing a refresher training. Some providers reported learning about the ESM-UBT device through word of mouth and during shifts at the hospital with other employees.

Several individuals described the need for regular refresher trainings for all OBGYN staff. Several midwives stated that doctors (postgraduates and MOs) are often posted to the OBGYN service and labor wards and then rotate; hence the need for recurrent trainings. Finally, a few providers mentioned that there was a dearth of staff to assist with PPH management hence instructing (untrained) personnel on the setup of the ESM-UBT device at the time of hemorrhage was sometimes stressful.

The most frequently reported barrier to ESM-UBT use was lack of knowledge on how to use the device; either because the provider had never been trained, was trained several months prior or had never seen an ESM-UBT device used. A few providers mentioned that they did not know how much fluid to instill into the ESM-UBT device and thus hesitated to use it. A small number of providers mentioned that they witnessed a UBT device placed in patients with cervical tears without uterine atony.

### ESM-UBT access/availability

Most health care workers stated that ESM-UBT devices were located on the labor or post-natal wards in labeled PPH kits that contain medications and devices commonly used to manage postpartum hemorrhage. Nearly half of the providers stated that ESM-UBT devices were readily available when needed and most reported they knew the exact location. A few providers described that in their experience ESM-UBT kits were not available on the labor ward or in the operating theatre when needed. All the interviewed providers stated that they could obtain ESM-UBT devices from other wards when necessary.

### Effect of prior experience

More than half of the providers described positive experiences with an ESM-UBT device, and several providers stated that they were more likely to use the ESM-UBT device after hearing about previous successes from other colleagues. Two providers reported they felt more comfortable using an ESM-UBT device when an individual who had prior experience with the device was working with them.

### Expansion of ESM-UBT availability

Another theme that emerged was the need for ESM-UBT training and device availability at peripheral hospitals. Several providers described that since ambulance services and blood products are scarce, hemorrhaging women often arrive at referral facilities too late to save them. Most individuals stated that expansion of the ESM-UBT program to lower levels of care would reduce morbidity and mortality.

### ESM-UBT adoption and uptake

A few individuals stated that their desire to save a life with an ESM-UBT device was a motivating factor. Approximately one third of providers described that the ability to potentially preserve a patient’s fertility and uterus was motivating. Nevertheless, the desire to gain surgical experience was mentioned as a potential reason for lower than expected rates of UBT adoption.

## Discussion

Postpartum hemorrhage continues to be a leading cause of maternal mortality worldwide with low-income countries disproportionately affected. Overall, 1 year after the introduction of the ESM-UBT device, the ESM-UBT device was perceived by all cadres as easy to use, accessible, and effective in saving lives. However similar to prior research in Sierra Leone, Senegal and Kenya several barriers to optimal uptake remain [28].

Several factors were identified that appeared to facilitate UBT use; perhaps one of the most important being the role of training. Given the nature of frequent provider turnover, regularly scheduled in-service trainings should be offered on a frequent basis. Pre-service training also represents a valuable opportunity to introduce UBT during the formative stages of a health care provider’s career. In addition, refresher trainings offer a way to ensure that providers remain knowledgeable and comfortable with use of the UBT device. This is especially important in lower volume facilities where uncontrolled postpartum hemorrhage may be seen less frequently. These findings are similar to previous studies regarding advanced structured resuscitation trainings such as Advanced Cardiac Life Support (ACLS) and *Neonatal Resuscitation* Program (NRP). Research has demonstrated that simulation-based interventions along with refresher courses had the greatest impact on skills retention [29]. Low-cost simulation and training have also been shown to be effective in low resource settings in the management of PPH [30–32] as well as other emergencies [33].

The interviews provided insights into factors that might motivate providers to use ESM-UBT devices; such as sense that the device might save a mother’s life or preserve a woman’s uterus. A similar sentiment was noted by Pendleton et al. in regard to health care providers’ motivation to use ESM-UBT devices in Kenya and Senegal [34]. The message that ESM-UBT devices may save mothers’ lives and avert hysterectomies should be highlighted in trainings and elsewhere.

Sharing UBT success stories may increase ESM-UBT device uptake given that providers reported increased comfort with the use after hearing about their colleagues’ previous successes. Potential avenues for sharing experiences include simple word of mouth as well as more formal settings such as lectures and hospital morning reports. Since providers expressed increased comfort with use of ESM-UBT devices when a colleague with previous ESM-UBT experience was present at the time of insertion, it may be beneficial to train additional providers from all cadres and establish mentorship programs/teams. In-country champions could also be utilized to provide encouragement and support as well as mentorship. Previous research has demonstrated the importance of ongoing dissemination of the findings to various actors in different levels of the health care system in order to ensure and maintain stakeholder buy-in for health programs in rural cities [21].

Expansion of UBT training and device availability at peripheral hospitals was an important factor that could facilitate use of UBT devices at these facilities and potentially contribute to decreases in morbidity and mortality. Given the lack of blood and surgical capabilities at many lower level hospitals as well as delays that often occur during the transfer process, use of UBT devices prior to transfer has the potential to reduce morbidity and mortality in women with uncontrolled PPH.

Several barriers to ESM-UBT use were identified. The finding that residents and medical officers at times feared anger and reprimand from senior physicians for using an ESM-UBT device highlights the need to enlist senior OBGYNs as champions. These senior-level OBGYN champions can address their colleagues’ resistance to change as well as enable provider fears to be directly discussed. As demonstrated in similar studies, local leadership is key in implementing interventions and the potential adaptation and scale up of programs and devices [35]. This may be critical in helping to alleviate the fear of possible retribution of senior health care providers. Lack of knowledge was another prominent barrier to ESM-UBT use. This lack of knowledge as well as waning of knowledge has the potential to impact the appropriate use of the device. Training all cadres of providers that attend deliveries to use UBT could ensure that more people in a facility are trained and therefore able to provide mentorship and support for UBT use during a PPH emergency.

Another described barrier was that ESM-UBT kits were not always available when needed. Ensuring that UBT devices are easily available and accessible at the time of an obstetrical emergency is essential as delays from trying to locate a kit may discourage use and could potentially impact hemorrhaging patients’ outcomes. As with previous studies, champions and/or specific PPH teams could address training needs as well as work with hospital staff to ensure availability and accessibility of UBT devices [35].

Our study findings are limited by our convenience sampling methodology and the potential for a desirability bias. Due to logistical constraints and staff scheduling, providers were recruited only if they met inclusion criteria and were willing and able to participate during the two-week interview period. An inherent and socio-culturally buttressed desire to not cause offence, particularly to those who are foreign and connected to the implementation of the intervention being studied, could have led to increased positive remarks. However, this seems unlikely to have introduced a significant bias as a broad range of perceptions were captured. Another potential limitation may stem from only conducting interviews at two hospitals, the National Referral Hospital and a smaller facility. This may have introduced a significant limitation as the patient populations and work environment of the facilities differ from one another.

## Conclusion

This study adds to the growing body of evidence that introduction and achievement of optimal uptake of new interventions, such as UBT, is complex. Facilitators to optimal use of the ESM-UBT device included formal training, refresher training, sharing success stories, and mentorship. Barriers included fear, lack of knowledge, inadequate supply, and fixed practice habits among senior doctors. As the ESM-UBT device is scaled, these factors should be incorporated into implementation strategies.

## Supplementary information

**Additional file 1.** ESM-UBT Mixed Methods Paper-Qualitative Interview Guide

## Data Availability

The dataset analyzed during the current study is not publicly available to protect the anonymity of the participants. Requests for access to the dataset used during the current study should be directed to the corresponding author.

## Abbreviations

ESM-UBT: Every Second Matters for Mothers and Babies- Uterine Balloon Tamponade (ESM-UBT)
PPH: Postpartum hemorrhage
