# Supplementary material for: A condom uterine balloon device among referral facilities in Dar Es Salaam: an assessment of perceptions, barriers and facilitators one year after implementation
Source: BMC Pregnancy Childbirth. 2020 Jan 13;20:34. doi: 10.1186/s12884-020-2721-9 (PMC6958625; doi:10.1186/s12884-020-2721-9)
Supplement: Supplementary file 1 — Additional file 1. ESM-UBT Mixed Methods Paper-Qualitative Interview Guide [file 12884_2020_2721_MOESM1_ESM.docx]

Appendix 1

**ESM-UBT Mixed Methods Paper-Qualitative Interview Guide**

**Introduction:**

We are research fellows with the Division of Global Health and Human Rights at the Massachusetts General Hospital in Boston, United States. We are here to learn about the circumstances surrounding postpartum hemorrhage management in this hospital, specifically facilitators and barriers to uptake of ESM-UBT. Thank you for taking the time out of your schedule to speak with us about your experiences. There are no right or wrong answers and you can stop this interview at any time. We will be audio recording this session so that we do not miss any of your comments. We are hoping that we may call you if we have any follow-up questions. Thank you again!

**General Information**

Provider Name:

Age:

Phone number:

Gender:

Cadre:

Years of Training:

Deliveries / month:

PPH cases / month personally cared for:

Your definition of PPH? Uncontrolled PPH?

Uncontrolled PPH cases in the past year:

# Hysterectomies performed/month (if doctor):

Length of time in your current position (if not one year or more, please stop interview):

Date of ESM-UBT training (if not trained within the past year please stop interview):

| **Code** | **Theme** | **Description** | **Examples** |
| --- | --- | --- | --- |
| F-Indv | Facilitator | Individual level facilitator | Bravery, prior experience, previous success |
| F-Indv-Train | Facilitator | Training |  |
| F-Indv-Save | Facilitator |  | Save lives/retain uterus |
| F-Indv-Brave | Facilitator | Bravery |  |
| F-Indv-Exp | Facilitator | Prior Experience | Assisted someone else, has seen UBT being used, |
| F-Indv-Prevsucc | Facilitator | Previous success | Successfully placed UBT |
| F-Indv-Oth | Facilitator | Other |  |
| F-Fac | Facilitator | Facility level facilitator | Kits available everywhere, incentives |
| F-Dev | Facilitator | Factors intrinsic to the kit | Ease of use |
| F-Pers | Facilitator | Personnel level | Teamwork, camaraderie etc |
| B-Indv | Barrier | Individual level barrier | Fear, apprehension |
| B-Indv-train | Barrier | Lack of training |  |
| B-Indv-fear | Barrier | Fear/Apprehension |  |
| B-Indv-Know | Barrier | Lack of Knowledge |  |
| B-Indv-diag | Barrier | Unsure of Diagnosis |  |
| B-Indv-Other | Barrier | Other |  |
| B-Fac | Barrier | Facility level barrier | Kits not available |
| B-Fac-kit | Barrier | Kit availability |  |
| B-Fac-transfer | Barrier | Transfer |  |
| B-Fac-Other | Barrier | Other |  |
| B-Dev | Barrier | Factors intrinsic to the kit | Difficult to use |
| Exec | Implementation | Factors pertaining to execution, implementation, roll out, administration | Too few trainings, high turnover, incentive structure |
| Exec-train | Training | Comments on training | Changes to training, more training, informal training, on the job training |
| Expan | Scale up/ Expansion | Comments on need at other facilities or advice on expansion | More training, expansion to peripheral facilities |
| Perc-Prov | Perception | Comments on providers’ perceptions | No negative comments |
| Perc-Pat | Perception | Comments on providers’ perceptions of patient perceptions | Comfortable, no complaints |
| PPH-M -prot | PPH Management | Comments on PPH management protocol | PPH protocol, oxytocin |
| PPH-M-UBT | UBT use | Comments on place of UBT in PPH management protocol | Confirming diagnosis before use, use after standard procedures have failed |
| PPH-M-Def |  | Definition |  |
| PPH-M-Case |  | Case Description |  |
| PPH-M-Other |  | Other |  |
| Fac-Sys | Facility System | Comments pertaining to staffing, management protocols, systems in place to intervention retrieval | Availability or lack of staff, |
| Surg | Surgery | Comments on surgical interventions related to PPH | Hysterectomies, theatre availability |
| Uter-rup | Uterine Rupture |  |  |
| Trad-med | Traditional Medications | Comments on natural medications | Teas, accelerating agents |
| Quotes | Quotations | Interesting quotation for paper |  |

| Hosp-Amana | Hospital | Amana |  |
| --- | --- | --- | --- |
| Hosp-Muhim | Hospital | Muhimbili |  |
| Cadre-MD | Cadre | Medical Doctor |  |
| Cadre- MD-Res | Cadre | Resident |  |
| Cadre- MD-OB | Cadre | OB/Specialist |  |
| Cadre-MW | Cadre | Midwife |  |
| Exp<5 | Experience | Less than 5 years |  |
| Exp>5 | Experience | Greater than 5 years |  |
| Gen-F | Gender | Female |  |
| Gen-M | Gender | Male |  |

**Coding Tree**

- Descriptive
  - Hospital
    - Amana (Hosp-Amana)
    - Muhimbili (Hosp-Muhim)
  - Cadre
    - OB (Cadre-OB)
    - GP (Cadre-GP)
    - Resident (Cadre-Res)
    - MW (Cadre-MW)
  - Exp
    - <5 (Exp<5)
    - >5 (Exp>5)
  - Gender
    - Female (Gen-F)
    - Male (Gen-M)
  - Demographics
- Facilitator
  - Individual
    - Save lives/retain uterus
    - Training
    - Bravery
    - Prior Experience
    - Previous Success
    - Other
  - Facility
  - Device
  - Personnel
- Barrier
  - Individual
    - Fear/apprehension
    - Knowledge
    - Unsure of diagnosis
    - Other
  - Facility
    - Kit availability
    - Transfer
    - Other
  - Device
  - Personnel
- Execution
  - General
  - Training
- Expansion
- Perception
  - Provider
  - Patient
- PPH Management
  - Definition
  - Protocol
  - Case Description
  - UBT use
  - Other
- Facility System (Fac-sys)
- Surgery (Surg)
- Uterine Rupture (Uter-rup)
- Traditional Medicines (Trad-med)
- Quotes (Quotes)
